# Contribution of the Alkylquinolone Quorum-Sensing System to the Interaction of *Pseudomonas aeruginosa* With Bronchial Epithelial Cells

**DOI:** 10.3389/fmicb.2018.03018

**Published:** 2018-12-18

**Authors:** Yi-Chia Liu, Farah Hussain, Ola Negm, Ana Carolina Paiva, Nigel Halliday, Jean-Frédéric Dubern, Sonali Singh, Sirina Muntaka, Lee Wheldon, Jeni Luckett, Paddy Tighe, Cynthia Bosquillon, Paul Williams, Miguel Cámara, Luisa Martínez-Pomares

**Affiliations:** ^1^ School of Life Sciences, University of Nottingham, Nottingham, United Kingdom; ^2^ Microbiology and Immunology Department, Faculty of Medicine, Mansoura University, Mansoura, Egypt; ^3^ Centre for Biomolecular Sciences, University of Nottingham, Nottingham, United Kingdom; ^4^ School of Medicine, University of Nottingham, Nottingham, United Kingdom; ^5^ School of Pharmacy, University of Nottingham, Nottingham, United Kingdom

**Keywords:** bronchial epithelial cells, *Pseudomonas aeruginosa*, quorum sensing, inflammation, pseudomonas quinolone signal

## Abstract

*Pseudomonas aeruginosa* causes infections in patients with compromised epithelial barrier function. Multiple virulence factors produced by *P. aeruginosa* are controlled by quorum sensing (QS) *via* 2-alkyl-4(1*H*)-quinolone (AQ) signal molecules. Here, we investigated the impact of AQs on *P. aeruginosa* PAO1 infection of differentiated human bronchial epithelial cells (HBECs). The *pqsA-E* operon is responsible for the biosynthesis of AQs including the 2-alkyl-3-hydroxy-4-quinolones, 4-hydroxy-2-alkylquinolines, and 4-hydroxy-2-alkylquinoline *N*-oxides as exemplified by pseudomonas quinolone signal (PQS), 2-heptyl-4-hydroxyquinoline (HHQ), and 2-heptyl-4-hydroxyquinoline *N*-oxide (HQNO), respectively. PQS and HHQ both act as QS signal molecules while HQNO is a cytochrome inhibitor. PqsE contributes both to AQ biosynthesis and promotes virulence in a PQS-independent manner. Our results show that PQS, HHQ, and HQNO were produced during PAO1 infection of HBECs, but no differences in growth or cytotoxicity were apparent when PAO1 and an AQ-negative Δ*pqsA* mutant were compared. Both strains promoted synthesis of inflammatory cytokines TNF-α, interleukin (IL)-6, and IL-17C by HBECs, and the provision of exogenous PQS negatively impacted on this response without affecting bacterial growth. Expression of *pqsE* and the PQS-independent PqsE-regulated genes *mexG* and *lecA* was detected during HBEC infection. Levels were reduced in the Δ*pqsA* mutant, that is, in the absence of PQS, and increased by exogenous PQS. These results support an AQ-independent role for PqsE during initial infection of HBEC by *P. aeruginosa* and for PQS as an enhancer of PqsE and PqsE-controlled virulence determinants and as an immunomodulator.

## Introduction

*Pseudomonas aeruginosa* is an opportunistic pathogen that causes acute infections in patients with ventilator-acquired pneumonia and is a major cause of chronic respiratory infections in patients with cystic fibrosis (CF) (Kipnis et al., 2006; Folkesson et al., 2012). *P. aeruginosa* employs at least two families of quorum-sensing (QS) signal molecules. The *N*-acyl homoserine lactones (AHLs), including *N*-(3-oxododecanoyl)-L-homoserine lactone (3-oxo-C12-HSL) and *N*-butanoyl-L-homoserine lactone (C4-HSL), are produced and sensed by the LasRI and RhlRI systems, respectively (Williams and Camara, 2009). The 2-alkyl-4(1*H*)-quinolones (AQs) including 2-heptyl-3-hydroxy-4(1*H*)-quinolone (PQS, pseudomonas quinolone signal) and its biosynthetic precursor 2-heptyl-4-hydroxyquinoline (HHQ) regulate gene expression through interactions with PqsR (MvfR) (Heeb et al., 2011; Ilangovan et al., 2013; Drees and Fetzner, 2015). PQS is also a ferric iron chelator involved in the iron-starvation response and virulence factor gene expression *via* both PqsR-dependent and PqsR-independent pathways (Bredenbruch et al., 2006; Diggle et al., 2007; Rampioni et al., 2016). *P. aeruginosa* also produces AQs such as 2-heptyl-4-hydroxyquinoline *N*-oxide (HQNO) that do not function as QS signals but are potent cytochrome inhibitors for both prokaryotic and eukaryotic cells (Heeb et al., 2011; Rampioni et al., 2016). HQNO also triggers bacterial cell autolysis beneficial for biofilm formation and antibiotic tolerance (Hazan et al., 2016). Most genes required for AQ biosynthesis (*pqsABCDE*) and response (*pqsR*) are located at the same genetic locus although *pqsH* and *pqsL* are distally located (Winsor et al., 2011). PqsA catalyzes the formation of anthraniloyl-CoA (Coleman et al., 2008) that is condensed with malonyl-CoA by PqsD to form 2-aminobenzoylacetyl-CoA (2-ABA-CoA). The latter is converted into 2-aminobenzoylacetate (2-ABA) *via* the thioesterase activity of PqsE (Drees and Fetzner, 2015). Although PqsE is not essential for AQ biosynthesis, it is required for the AQ-independent production of several virulence factors that contribute to biofilm maturation including pyocyanin, rhamnolipids, and lectin A (Rampioni et al., 2016). Defects in AQ biosynthesis or sensing causes attenuation of *P. aeruginosa* in plant and animal infection models (Deziel et al., 2005; Rampioni et al., 2010; Dubern et al., 2015). *P. aeruginosa* PAO1 *pqsA* and PA14 *pqsR* mutants were both severely attenuated in murine models of infection (Deziel et al., 2005; Rampioni et al., 2010). Inhibition of AQ signaling also supports the importance of PQS in both acute and persistent *P. aeruginosa* infections in mice (Starkey et al., 2014).

PQS not only positively regulates the expression of *P. aeruginosa* virulence factors (McKnight et al., 2000; Diggle et al., 2003; Reis et al., 2011; Jimenez et al., 2012; Rampioni et al., 2016) but also displays immunomodulatory properties. In peripheral blood mononuclear cells, purified PQS inhibits T-cell proliferation after stimulation with either concanavalin A or CD3/CD28 and inhibits interleukin (IL)-2 production after stimulation with concanavalin A (Hooi et al., 2004). PQS also inhibited IL-12 production and T-cell activating capacity in mouse bone marrow-derived dendritic cells (Skindersoe et al., 2009). Addition of PQS and HHQ to cell-free supernatants from a PA14 Δ*pqsA* mutant (i.e., a strain unable to synthesize AQs) inhibited TNF-α and IL-6 production by murine macrophages through the NF-κB pathway (Kim et al., 2010a,b). PQS directly downregulates hypoxia-inducible factor 1 (HIF-1α) levels in human epithelial cells through a 26S proteasome-dependent mechanism (Legendre et al., 2012).

AQs including PQS and HHQ have been found in the sputum, distal airways, blood, and urine of *P. aeruginosa*-infected CF patients and shown to correlate with clinical status (Collier et al., 2002; Barr et al., 2015, 2017) demonstrating that AQ-dependent QS is functional during chronic infections including acute exacerbations.

Airway epithelial cells provide a physical barrier through the production of mucus and antimicrobials and modulate immune activation *via* secretion of chemical mediators (Whitsett and Alenghat, 2015). Many *P. aeruginosa* virulence factors regulated *via* AQ-dependent signaling affect respiratory defence mechanisms. Purified elastase (LasB) disrupts airway epithelial barrier function *via* the degradation of the tight junction proteins zonula occludin 1 and 2 (ZO-1 and ZO-2) in type II alveolar epithelial cells and Madin-Darby canine kidney (MCDK) cell monolayers (Azghani, 1996). Purified rhamnolipids and pyocyanin disrupt tight junctions and inhibit mucociliary beating and secretion of mucus glycoconjugates from human and sheep trachea/bronchial epithelial cells (Graham et al., 1993; Lau et al., 2004). However, whether AQs influence the infection of human bronchial epithelial cells (HBECs) by *P. aeruginosa* is unknown.

This study demonstrates that AQs are produced during *P. aeruginosa* infection of HBECs. However, no differences in the growth and cytotoxicity of a *P. aeruginosa pqsA* mutant that cannot produce AQs were observed when compared with the wild type. In addition, no differences in pro-inflammatory cytokines in response to infection were detected. Addition of exogenous PQS significantly enhanced *pqsA* promoter activity and reduced the inflammatory response to *P. aeruginosa* without affecting bacterial growth or cytotoxicity. Finally, transcription of *pqsE* and the PqsE-regulated virulence factors *mexG* and *lecA* occurred in the AQ-negative *pqsA* mutant, albeit at lower levels than the isogenic PAO1 parent strain. Transcription of *pqsE, mexG,* and *lecA* was upregulated by the addition of PQS. These results demonstrate basal expression of *pqsE* and PqsE-regulated virulence genes during infection of HBECs that can be upregulated by AQs, and for PQS as immunomodulator of these cells during *P. aeruginosa* infection.

## Materials and Methods

### Bacterial Strains, Cells, and Growth Conditions

*P. aeruginosa* PAO1 (PAO1 Lausanne sub-line), a PAO1 Δ*pqsA* isogenic mutant, (see below) and the *pqsA*-reporter strain PAO1 *pqsA::lux* (Fletcher et al., 2007) were used in this study. Bacteria were grown on LB agar or in LB broth at 37°C, 200 rpm. The HBEC line Calu-3 was obtained from the American Type Culture Collection (ATCC no. HTB-55) and used between passages 19 and 35. Undifferentiated Calu-3 cells were maintained in minimum essential medium α GlutaMAX^TM^ I (MEM, Life Technologies, UK) supplemented with 10% fetal bovine serum (Sigma-Aldrich, UK). Calu-3 cells were differentiated at the air-liquid interface (Calu-3-ALI) as previously described (Zhu et al., 2010; Stewart et al., 2012). Briefly, Calu-3 cells (10^5^) were seeded on 0.4 μm pore size transwell inserts (Corning Life Sciences, NE) and fed with 500 μl of culture medium in the lower chamber every other day for 21 days. Calu-3-ALI cultures exhibited epithelial integrity and polarity featured with the expression of tight junction protein zonula occludin 1 (ZO-1), cilia, and mucus production at the apical surface (Figure S1).

### Generation of Δ*pqsA*

An in-frame deletion of *pqsA* constructed in PAO1 that combined a 410-bp upstream fragment of the *pqsA* gene with a 423-bp downstream fragment was generated by overlap extension polymerase chain reaction (PCR) using *P. aeruginosa* PAO1 chromosomal DNA as template. The upstream 410-bp fragment was amplified using the forward primer pqsAf1 which carries a *Xba*I restriction site 5′-ATATCTAGACGCCTCGAACTGTGAGATTT-3′, and the reverse primer pqsAr1 containing the first 12 nucleotides of the *pqsA* gene and an overhanging end containing the last 15 nucleotides of *pqsA* gene (underlined) 5′-TCATGCCCGTTCCAATGTGGACATGACAGAACG-3′; the downstream 423-bp fragment was amplified using forward primer pqsAf2 containing the last 15 nucleotides of *pqsA* gene and an overhanging end containing the first 12 nucleotides of *pqsA* with its 3 upstream nucleotides (underlined) 5′-GTCATGTCCACATTGGAACGGGCATGTTGATTCAGG-3′ and the reverse primer pqsAr2 which carries a *Hin*dIII restriction site 5′-TATAAGCTTACTCGCTGTCCACTTCCAAT-3′. To perform the overlap extension PCR, a second PCR was performed using the 410 and 423-bp fragments as templates and the primers pqsAf1 and pqsAr2. The final PCR product was cloned using the *Xba*I and *Hin*dIII restriction sites into the suicide vector, pME3087, resulting in plasmid pYCL1. The Δ*pqsA* deletion was generated by allelic exchange using pYCL1 as described (41). Bacterial strains used to generate the Δ*pqsA* mutant are listed in Table S1. The Δ*pqsA* deletion was verified by nucleotide sequencing and phenotypic analysis (see Figures S2A–D).

### Infection of Human Bronchial Epithelial Cells

Overnight bacterial cultures in LB broth were normalized to OD_600_ 0.1 in fresh LB broth and cultured for 4 h. Bacterial cells were washed twice with PBS containing Ca^2+^ and Mg^2+^ (PBS-Ca^2+^/Mg^2+^) and suspended in PBS-Ca^2+^/Mg^2+^ at a concentration of 3.5 × 10^8^ CFU/ml for multiplicity of infection (MOI) 50 or 3.5 × 10^6^ CFU/ml for MOI 0.5. Bacterial suspensions (100 μl) were added to the apical surface of Calu-3-ALI cultures and incubated at 37°C, 5% CO_2_ for different time periods as stated in the text. Controls were Calu-3-ALI cultures treated with 100 μl of PBS-Ca^2+^/Mg^2+^ (untreated) and cell culture medium inoculated with bacteria (*P. aeruginosa* only). In some instances, Calu-3-ALI cultures were treated with 2-heptyl-3-hydroxy-4(1*H*)-quinolone (PQS, synthesized in house (Diggle et al., 2003) dissolved in dimethyl-sulfoxide (DMSO) to a final concentration of 20 and 40 μM 1 h prior to infection. Control cultures were treated with equivalent amount of DMSO.

### Quantification of 2-alkyl-4(*1H*)-Quinolones in *P. aeruginosa*-Infected Calu-3-ALI Cultures

Extraction and quantification of AQs was based on a previously described method (Ortori et al., 2011). Briefly, culture medium (400 μl) was added to the transwell inserts, and the medium in the transwell insert and lower chambers was collected from cultures. Cell-free supernatants were prepared by filtration through a 0.2-μm polytetrafluoroethylene (PTFE) syringe filter. From each supernatant, a 200-μl sample was spiked with 10 μl of an internal standard solution (10 μM uniformly deuterated PQS (d4-PQS in MeOH)), and extracted three times with 0.5 ml of acidified ethyl acetate (0.1% (v/v) AcOH in EtOAc) (Sigma-Aldrich, UK). Combined organic extracts from each sample were dried under vacuum (Jouan RC10 Speedvac, Thermo Scientific) and re-dissolved in 50 μl of MeOH prior to liquid chromatography mass spectrometry (LC-MS/MS) analysis. Analysis was conducted using reversed phase chromatography (Phenomenex Gemini C18 column (3.0 μm, 50 × 3.0 mm) installed in a Shimadzu Series 10AD VP LC system), in tandem with an Applied Biosystems QTRAP 4000 hybrid triple quadrupole-linear ion trap mass spectrometer. Calibration samples were prepared by spiking 200 μl of sterile media with 50 μl of methanolic calibration mix containing HHQ, PQS, and HQNO at concentrations from 1 to 100 nM, and extracting with ethyl acetate.

### Assessment of *pqsA* Promoter Activity During Infection of Calu-3-ALI Cultures

Differentiated Calu-3-ALI cultures were infected with WT and PAO1 *pqsA::lux* reporter strain in the presence and absence of exogenous PQS as described above. The wells were visualized using a Hamamatsu luminometer and Wasabi software. Luminescence was quantified using Image J.

### Quantification of Bacterial Growth During Infection

For quantification of cell-associated bacteria, Calu-3-ALI cultures were lysed with 250 μl of 0.1% Triton X-100 (Sigma-Aldrich, UK) and homogenized by repeated pipetting. Serial dilutions were prepared in PBS-Ca^2+^/Mg^2+^ and 100 μl of each dilution was plated on LB agar plates. Colony-forming unit (CFU) was calculated by multiplying the number of colonies by the corresponding dilution factor. For quantification of bacteria in the lower chamber, the basal culture media were collected, subjected to serial dilutions, and CFU calculated as described above.

### Reverse Phase Protein Microarray

Reverse phase protein microarray analysis (Spurrier et al., 2008) was performed to evaluate the presence of β-actin, nucleoporin 98-kDa (NUP98), human cilia-associated tubulin IV, human mucin MUC5AC, human zonula occludin-1 (ZO-1) protein, and human E-cadherin by their correspondent antibodies on lysates of Calu-3-ALI cultures post infection (Life Technologies, USA; Sigma-Aldrich, UK; Cell Signalling technology, UK). Calu-3-ALI cultures with or without WT or Δ*pqsA* infection at 3 and 6 hpi were lysed with 70 μl of RIPA buffer (Thermo Scientific Pierce, UK) supplemented with protease and phosphatase inhibitors (Thermo Scientific Pierce, UK). Data were analyzed using RPPanalyzer software written in R. Evaluation of cytotoxicity was based on the loss of fluorescence signal quantified as arbitrary fluorescence units (AFUs).

### Cytotoxicity Assay

Cytotoxicity in undifferentiated Calu-3 cultures was assessed using the lactate dehydrogenase (LDH) colorimetric assay according to the protocol provided by the manufacturer (Roche Applied Sciences, UK). Calu-3 cells were seeded in 24-well tissue culture plates (Corning Life Sciences, Netherlands) at a density of 100,000 cells per well and cultured for 3 days. On the day of infection, culture medium was replaced with minimum essential medium α GlutaMAX I without fetal bovine serum. Cells were infected with WT PAO1 or Δ*pqsA* in PBS-Ca^2+^/Mg^2+^ at MOI 50 for 3 and 6 h. Cells incubated with PBS-Ca^2+^/Mg^2+^ were used as negative controls. Cell-free supernatants were collected and centrifuged to remove cellular debris and bacteria. Supernatants from uninfected cells were used as negative controls and those collected from Calu-3 cells lysed with 2% of Triton X-100 represented the 100% cell death positive control. The percentage of cell death was calculated by comparison with the optical density (OD) readings from 100% cell death controls.

### Western Blot Analysis

Calu-3-ALI cultures were lysed as above and processed for Western blot analysis to detect actin using mouse monoclonal anti-β-actin (1 μg/ml, Sigma-Aldrich, UK) followed by HRP-conjugated goat anti-mouse secondary antibody (1:10,000 dilution, Sigma-Aldrich, UK). Antibody binding was detected using the enhanced chemiluminescence (ECL) detection reagent (GE Healthcare, UK).

### Quantitative Real-Time PCR

RNAs were extracted from Calu-3-ALI cultures using the Absolutely RNA Miniprep Kit (Stratagene, UK) according to manufacturer’s instructions. The quantity and quality of RNA were determined using a NanoDrop 2000 spectrophotometer (Thermo Scientific, UK). First cDNA strand was synthesized from 3 μg of total RNA using random hexamers and Moloney Murine Leukemia Virus (M-MLV) Reverse Transcriptase (Life Technologies, UK) according to manufacturer’s instructions (Life Technologies, UK). Reverse transcript negative controls were also performed.

Quantitative real-time PCR (qPCR) was performed to determine transcription of genes encoding granulocyte-macrophage colony-stimulating factor (GM-CSF), TNF-α, IL-6, IL-17C, and IL-8, according to the MIQE guidelines (Bustin et al., 2009). Primers are shown in Table S2. Primers were tested for efficiency *via* construction of a standard curve performed with templates in 4-log serial dilutions and efficiencies between 99 and 110% were considered appropriate. 2x SYBR Green I Master Mix with 2.5 mM MgCl_2_ and ROX as reference dye were used (Stratagene, UK). Each sample was tested in triplicate and the thermal cycling setting was as follows: 95°C for 3 min, 40 cycles of 95°C for 10 s, and 72°C for 30 s. The relative gene expression changes between samples were calculated using the ΔΔCq method using hypoxanthine-guanine phosphoribosyltransferase (HPRT) as reference. Analysis was carried out with the program MxPro PCR software Mx3005P (Stratagene, UK). Total mRNA was prepared at specific times.

### Reverse Transcription PCR

For reverse transcriptase PCR (RT-PCR), total RNA was extracted from Calu-3 cell culture infected with *P. aeruginosa* PAO1 wild type or ∆*pqsA* using a Qiagen RNeasy extraction kit (Qiagen). cDNA synthesis was performed from 1 μg of total purified RNA by using random hexamer primers and GoScript reverse transcriptase (Promega). A total of 50 ng of resulting cDNA was PCR-amplified using Expand High Fidelity PCR System (ROCHE) and primers *pqsE*RT For and *pqsE*RT Rev (for *pqsE*), *pqsA*RT For and *pqsA*RT Rev (for *pqsA*), *oprL*RT For and *oprL*RT Rev (for *oprL*), *mexG*RT For and *mexG*RT Rev (for *mexG*), and *lecA*RT For and *lecA*RT Rev (for *lecA*) (Table S3). After 5 min of denaturation at 95°C, the following reaction cycle was used for 35 cycles: 95°C for 30 s, 55°C for 30 s, and 72°C for 1 min. The PCR products were analyzed on a 2% (w/v) agarose gel and stained with Tracklt Cyan/Orange (Invitrogen).

### Cytokine Quantification

The levels of GM-CSF in the basal culture media were quantified using a Capture ELISA following the manufacturers’ instructions (R&D, UK). The detection limit for this assay ranges between 10 and 1000 pg/ml. Simultaneous quantification of the levels of IL-6 and TNF-α in the basal media was conducted using the FlowCytomix Simplex kit bead-based immunoassay (eBioscience, UK) according to the manufacturers’ instructions. Samples were analyzed by flow cytometry (Beckman Coulter FC500) and the concentrations were analyzed using the FlowCytomix Pro 2.4 software (eBioscience, UK). The detection limit for these assays ranges between 20 and 20,000 pg/ml. In later experiments, a multiplex system (R&D systems) was employed to quantify levels of TNF-α, IL-6, IL-17C, IL-1β, IL-1α, and IL-33. Samples were analyzed using a Bio-Rad Bio-Plex 200 system.

### Statistical Analysis

Statistical analyses were performed in GraphPad Prism. Significance was assessed using unpaired t-tests when comparing two groups or one-way analysis of variance (ANOVA) with Tukey’s *post hoc* test when comparing more than two groups.

## Results

### AQs Are Produced During Infection of Differentiated HBECs by *P. aeruginosa*

**Figure 1:**
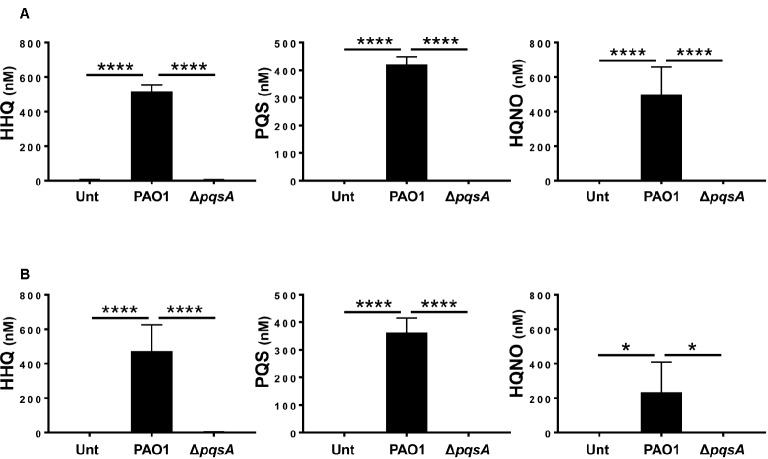
AQs are produced in Calu-3-ALI cultures infected with *P. aeruginosa*. Concentrations of HHQ, PQS, and HQNO in transwell inserts and in the basal medium of Calu-3-ALI cultures after PAO1 or Δ*pqsA* infection for 6 h were determined by LC-MS/MS. HHQ, PQS, and HQNO were detectable in the transwell inserts **(A)** and in the lower chamber **(B)** from Calu-3-ALI cultures infected with PAO1 at MOI 50. AQs were virtually undetectable in Calu-3-ALI cultures infected with Δ*pqsA*. No PQS, HHQ, and ΗQNO were detected in uninfected Calu-3-ALI cultures (Unt). Infections with PAO1 are shown in black bars; infections with Δ*pqsA* are shown in white bars. The results were obtained from four independent experiments and each experiment was done in triplicate. Values in **(A)** have not been corrected for the dilution factor (1:5); hence, the concentration of AQs in the samples is five times higher. *p* was calculated by one-way ANOVA. *p < 0.05, ****p < 0.0001.

To determine whether AQs are produced during infection of differentiated HBECs, differentiated Calu-3 cells in air-liquid interface cultures (Calu-3-ALI; Figure S1) were infected with PAO1 or the isogenic AQ-negative Δ*pqsA* mutant (Figure S2) at MOI 50 and AQs in the infected cultures were quantified by LC-MS/MS as described in Materials and Methods. The results show that HHQ, PQS, and HQNO are all detectable in Calu-3-ALI cultures infected by PAO1 at concentrations of 2.5 ± μM, 2 ± μM, and 2.5 ± μM, respectively, in the upper chamber, and 600 ± nM, 350 ± nM and 200 ± nM in the lower chamber. In contrast, no AQs were detected in uninfected cultures or cultures infected with the Δ*pqsA* mutant (Figure 1). These results demonstrate production of AQs during PAO1 infection of Calu-3-ALI cultures in a *pqsA*-dependent manner.

### PAO1 and the Δ*pqsA* Mutant Display Similar Pathogenic Characteristics During Infection of Calu-3-ALI Cultures

**Figure 2:**
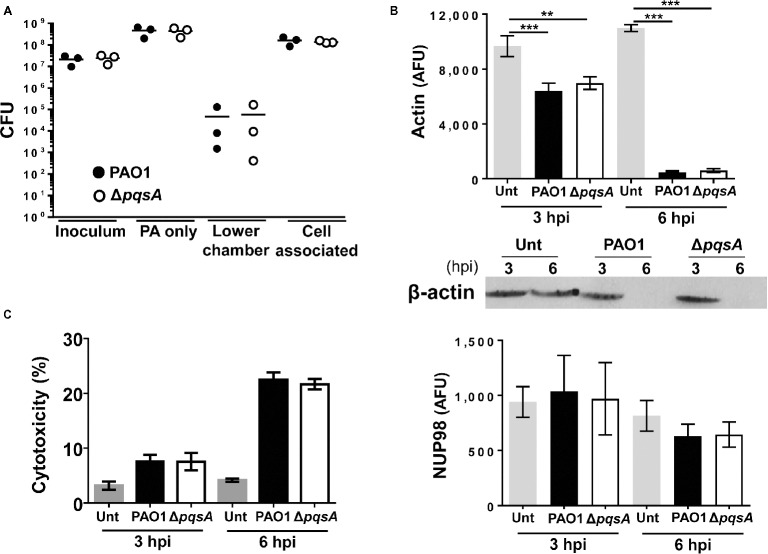
Comparison of PAO1 and Δ*pqsA* growth and cytotoxicity during infection of Calu-3-ALI cultures. **(A)**. Bacterial growth upon infection of Calu-3-ALI cultures with PAO1 and Δ*pqsA*. Bacteria derived from three compartments of Calu-3-ALI cultures infected at MOI 50 including cell-associated bacteria, bacteria in the lower chamber, and bacteria in cell-free wells were quantified as CFU at 6 hpi. Data were obtained from three independent experiments and each experiment was performed in duplicate. Each data point represents the mean of a single experiment. (PAO1: black circles; Δ*pqsA* white circles). **(B)**. Reverse protein microarray analysis of infection-induced cytotoxicity. Calu-3-ALI cultures were infected with PAO1 and Δ*pqsA* at MOI 50 and lysates generated at 3 and 6 hpi were subjected to protein microarray analysis to determine the levels of actin (upper panel) and NUP98 (lower panel). Specificity of the actin signal was confirmed using immunoblot (middle panel) **(C)** PAO1- and Δ*pqsA*-induced cytotoxicity upon infection of undifferentiated Calu-3 cells. Supernatants from Calu-3 cells infected with PAO1 and Δ*pqsA* at MOI 50 for 3 and 6 hpi were collected and tested for LDH levels. Data were obtained from three independent experiments and each experiment was performed in duplicate. (AFU, arbitrary fluorescence unit; Unt, untreated). *p* was assessed by one-way ANOVA. **p < 0.005, ***p < 0.001.

To investigate the contribution of AQs to *P. aeruginosa* growth and cellular damage during infection, Calu-3-ALI cultures were infected with PAO1 or the Δ*pqsA* mutant at MOI 50 and cell-associated bacteria, and bacteria in the lower chamber were quantified after 6 hpi. Growth of Δ*pqsA* was similar to that of PAO1 (Figure 2A, cell-associated) and consistent with their growth profiles in wells containing medium only (Figure 2A, PA only) or when cultured in LB broth (Figure S2A). The bacterial counts in the lower chamber at 6 hpi represented a minor proportion of the total bacterial population and, in agreement with the data obtained from the quantification of cell-associated bacteria, no significant differences were observed between PAO1 and Δ*pqsA-*infected cultures (4.7 ± 7.2 × 10^4^ (SD) and 5.8 ± 9.2 × 10^4^ (SD), respectively). These results indicate that the AQ-QS system is not required for promoting bacterial survival or growth during infection of Calu-3-ALI cultures at high MOI.

To investigate whether AQs contribute to *P. aeruginosa* virulence by altering its ability to cause cell damage and, in turn, promote breakdown of the epithelial barrier, the degradation of Calu-3 cell actin after infection with either PAO1 or Δ*pqsA* was quantified using a microarray-based assay and Western blot analysis. This methodology was chosen because of the variable infection patterns observed during infection ( Figure S3). Initial confocal analysis failed to provide a consistent account of the infection process with areas within the same infection displaying differential levels of cell damage and bacterial growth (Figure S3). PAO1 infection caused significant loss of the actin fluorescence signal at both 3 and 6 hpi compared with untreated cells and no differences were observed between PAO1 and Δ*pqsA*-infected cultures (Figure 2B). A reduction in the actin-specific signal occurred alongside reduction in the levels of ZO-1, E-cadherin, HSP90, mucin MUC5AC, and type IV β-tubulin in Calu-3-ALI cultures (Figure S4). Levels of the nuclear protein NUP98 were similarly maintained at 3 and 6 hpi after infection with both Δ*pqsA* and PAO1 (Figure 2B). The comparable loss of cellular proteins during PAO1 and Δ*pqsA* mutant infections of Calu-3-ALI cultures was in line with the levels of lactate dehydrogenase produced by undifferentiated Calu-3 cells after infection with these strains (Figure 2C). These results indicate that AQs do not contribute to *P. aeruginosa*-induced cytotoxicity in Calu-3-ALI cultures under these experimental conditions.

The production of AQs by *P. aeruginosa* in laboratory culture flasks is maximal during the stationary phase of growth, suggesting that AQ-dependent QS may play a role only at a late stage post-infection (Diggle et al., 2003). We postulated that the cell damage caused by PAO1 infection at high MOI provided a narrow window of opportunity to assess the contribution of ΑQs to lung epithelium colonization by *P. aeruginos*a. Hence, the infection assay was modified to model chronic infection and Calu-3-ALI cultures were infected at MOI 0.5 with both PAO1 and Δ*pqsA* for 6, 9, 12, and 24 h (Figure S5). PAO1 and Δ*pqsA* displayed similar growth when infections were performed at low MOI. Bacterial growth was detected both in the cell-associated fraction and in the lower chamber. Together, these data show that AQs do not influence PAO1 growth during high and low MOI infection of Calu-3-ALI cultures.

### Comparable Induction of Pro-inflammatory Responses by PAO1 WT and Δ*pqsA* in Calu-3-ALI Cultures

**Figure 3:**
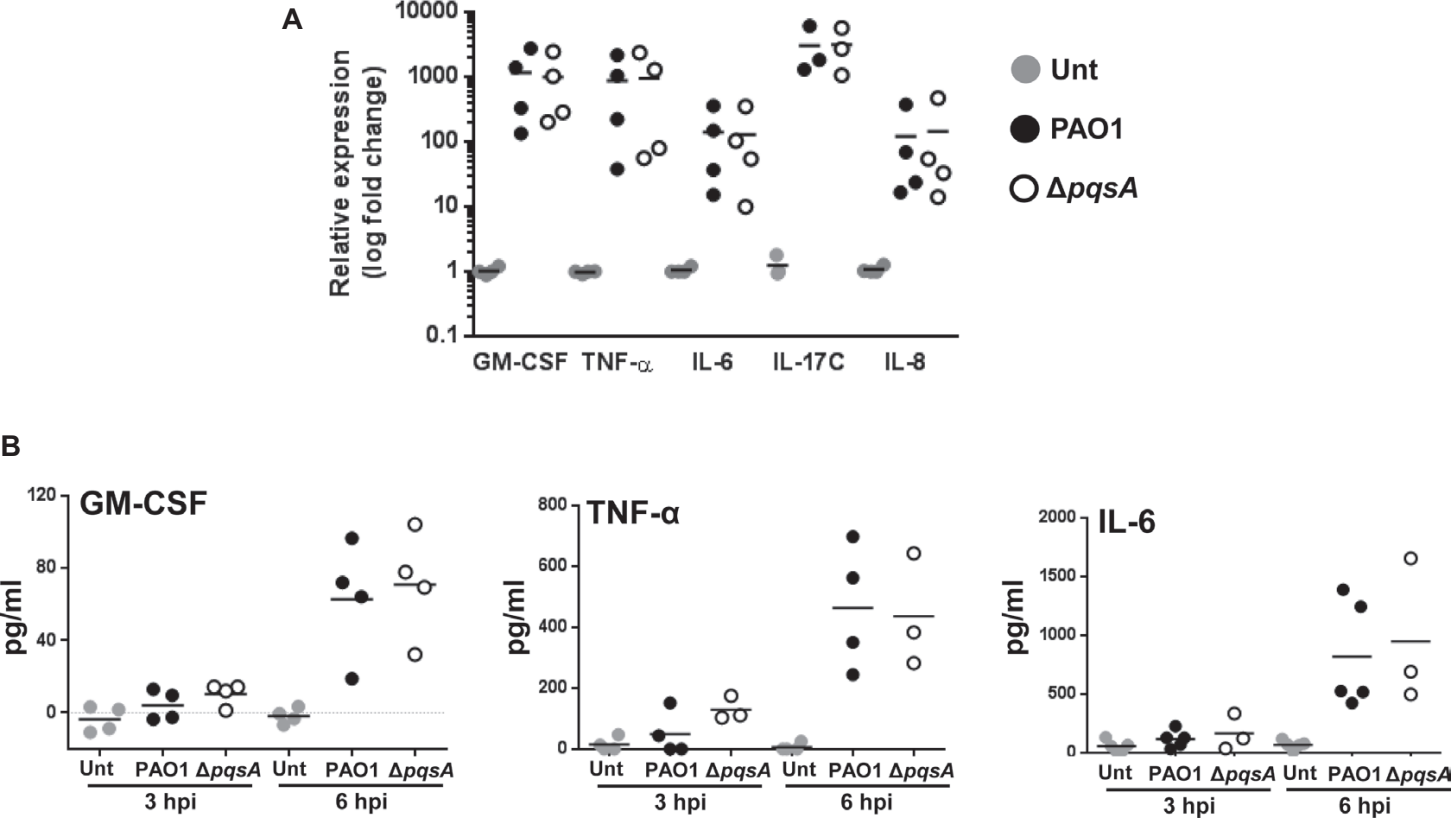
Induction of pro-inflammatory cytokines in Calu-3-ALI cultures following infection with PAO1. **(A)**. Relative expression of mRNAs coding for GM-CSF, TNF-α, IL-6, IL-17C, and IL-8 upon infection at MOI 50 of Calu-3-ALI cultures with PAO1 and Δ*pqsA* at 2 hpi assessed by qPCR. **(B)**. Levels of GM-CSF, TNF-α, and IL-6 in the lower chamber of Calu-3-ALI cultures infected with PAO1 and Δ*pqsA* at 3 and 6 hpi. Data were obtained from four independent experiments and each experiment was performed in duplicate.

To examine the impact of the AQ system on the induction of innate immune responses in airway epithelial cells in response to *P. aeruginosa* infection, the transcriptional expression and production of pro-inflammatory mediators by Calu-3-ALI cultures after infection with PAO1 or Δ*pqsA* were determined by quantitative real-time PCR and immuno-assays, respectively. Comparable upregulation of GM-CSF, TNF-α, IL-6, IL-17C, and IL-8 transcripts by PAO1 and Δ*pqsA* was found at 2 hpi (Figure 3A). In agreement with these observations, GM-CSF, TNF-α, and IL-6 were detected at similar levels in supernatants of PAO1 and Δ*pqsA* infected cultures (Figure 3B). Hence, lack of AQ synthesis does not affect production of pro-inflammatory cytokines by Calu-3-ALI cultures during *P. aeruginosa* PAO1 infection.

### Addition of Exogenous PQS Attenuates the Inflammatory Response of Calu-3-ALI Cultures to PAO1 Infection

*P. aeruginosa*-infected CF lung is exposed to AQs that could precondition epithelial cells to *P. aeruginosa* infection (Collier et al., 2002; Barr et al., 2015). Also, the addition of PQS to bacterial supernatants from a *pqsA* mutant has been shown to inhibit TNF-α and IL-6 production by mouse macrophages (Kim et al., 2010a,b) and purified PQS suppressed the production of HIP-1α in human epithelial cells with a cystic fibrosis transmembrane conductance regulator (CFTR) mutation (IB3-1) (Legendre et al., 2012). Therefore, it was important to determine whether exogenous PQS could alter activation of HBEC in response to infection with *P. aeruginosa*.

**Figure 4:**
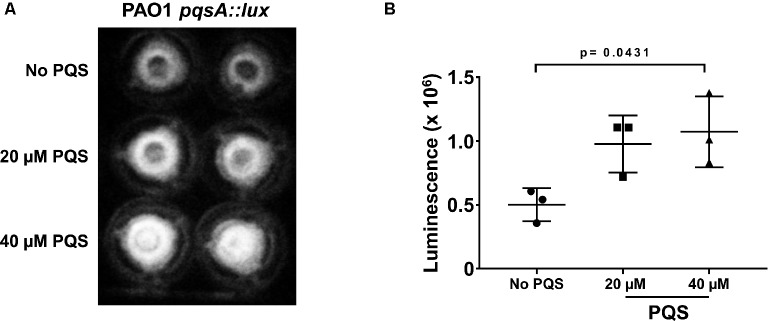
Exogenous PQS increases activation of the *pqs*A promoter during infection of Calu-3-ALI cultures. Calu-3-ALI cultures were infected with PAO1-*pqsA::lux* at MOI 50 in the presence of PQS (20, 40 μM) or the solvent DMSO (No PQS). Bioluminescence was visualized at 4 hpi in the transwells using a luminometer and quantified using Image J. **(A)** Representative images of luminescence in transwells upon infection with PAO1-*pqsA::lux* in the absence and presence of PQS in duplicate. **(B)** Analysis of luminescence intensity upon infection with PAO1-*pqsA::lux* from three independent infections performed in duplicate. Data were analyzed using one-way ANOVA with Tukey’s *post hoc* test.

In the first instance, exogenous PQS was tested for its ability to influence gene expression in *P. aeruginosa* during infection of Calu-3-ALI cultures. For this, *pqsA* promoter activity in the presence and absence of exogenous PQS (20 and 40 μM) was investigated using a *pqsA* promoter reporter strain. *pqsA* promoter activity was detected 4 h post-infection (Figure 4) and was significantly enhanced by addition of exogenous PQS (40 μM), suggesting that exogenous PQS can regulate gene expression in *P. aeruginosa* PAO1 during infection of Calu-3-ALI cultures.

**Figure 5:**
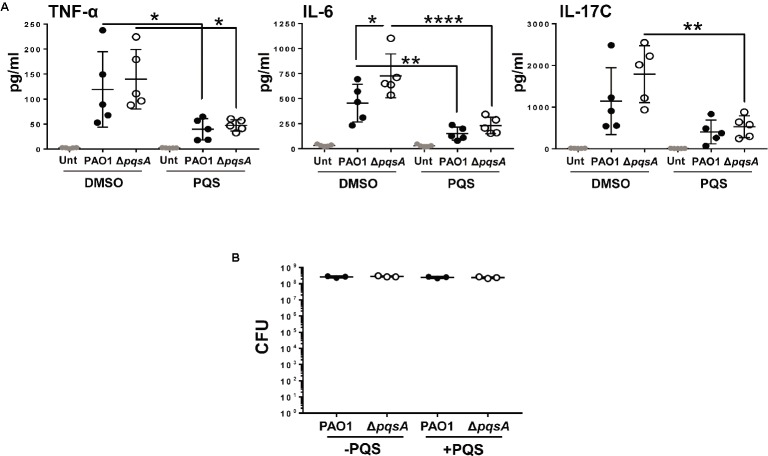
Reduced production of TNF-α, IL-6, and IL-17C, during PAO1 and Δ*pqsA* infection of Calu-3-ALI cultures in the presence of exogenous PQS. **(A)**. Calu-3-ALI cultures exposed to PQS (40 μM) or DMSO were infected with WT or Δ*pqsA* at MOI 50 and at 3 hpi, supernatants were collected and analyzed for levels of TNF-α, IL-6, and IL-17C. Data were obtained from five independent experiments in duplicate. *p* was assessed by one-way ANOVA with Tukey’s *post hoc* test. *p < 0.05, **p < 0.01, ****p < 0.0001. **(B)**. PQS does not influence bacterial growth during infection of Calu-3-ALI cultures. Cell-associated bacteria from Calu-3-ALI cultures exposed to PQS or DMSO and infected with PAO1 or Δ*pqsA* were quantified at 6 hpi. Data were obtained from three independent experiments in duplicate.

Next, Calu-3-ALI cultures were pre-treated with 40 μM PQS and then infected with PAO1 or Δ*pqsA*. This concentration was chosen because it significantly increased the activity of the *pqsA* promoter. Supernatants were collected at 3 hpi and tested for the presence of IL-17C, IL-6, TNF-α, IL-1β, IL-1α, and IL-33. Levels of IL-1β, IL-1α, and IL-33 were below the detection limit of the assay (data not shown). TNF-α, IL-6, and IL-17C were readily detected in supernatants from infected cultures (Figure 5A). In the absence of exogenous PQS, there was a tendency for increased production of IL-6 and IL-17C in the Δ*pqsA*-infected cultures compared to PAO1 with this trend becoming significant in the case of IL-6. Exogenous PQS reduced TNF-α and IL-6 production in WT and Δ*pqsA*-infected cultures and IL-17C production in the Δ*pqsA*-infected cultures. The growth of WT and Δ*pqsA* was similar in the presence and absence of exogenous PQS (Figure 5B). Exogenous PQS did not affect infection-mediated cytotoxicity (Figure S6A). These findings further corroborate the immunosuppressive role of PQS during *P. aeruginosa* infection (Hooi et al., 2004; Skindersoe et al., 2009; Kim et al., 2010b) albeit at doses probably not achieved in this PAO1-Calu-3-ALI infection model.

### Transcription of the PqsE-Controlled Virulence Factors *mexG* and *lecA* Occurs Independent of AQ’s Production During Infection of Calu-3-ALI Cultures

**Figure 6:**
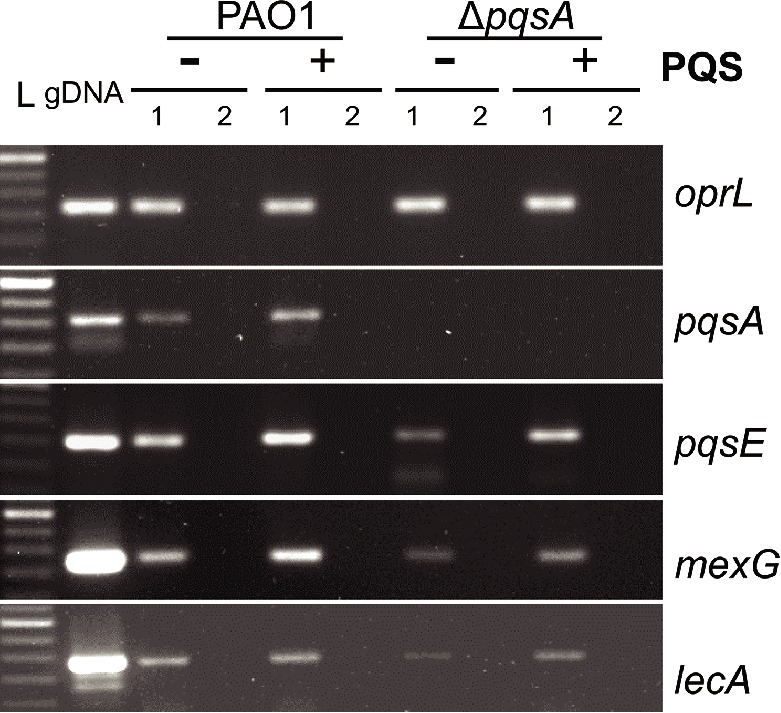
Analysis of *pqsA*, *pqsE*, *mexG,* and *lecA* transcription in WT and Δ*pqsA* Calu-3-ALI-infected cultures in the presence and absence of exogenous PQS. Expression of *pqsA*, *pqsE*, *mexG,* and *lecA* during infection of Calu-3-ALI cultures with PAO1 and Δ*pqsA* PAO1 at 3 hpi was assessed by RT-PCR. Calu-3-ALI cultures exposed to PQS (40 μM) or DMSO were infected at MOI 50 and total RNA prepared at 3 hpi. A 250-bp DNA region within the *pqsA* gene and a 200-bp DNA region within *pqsE, oprL*, *mexG,* and *lecA* genes were amplified from PAO1 genomic DNA (positive control); 1, cDNA; 2, corresponding RNA (negative control). L, 50 bp DNA ladder. Data are representative from 2 independent experiments.

Following the analysis of PAO1 growth, cytotoxicity, and immunostimulatory ability in the presence and absence of endogenous and exogenous PQS, it was important to determine the expression of selected AQ-regulated virulence factors during PAO1 infection of Calu-3-ALI cultures under these conditions. Since PqsE has a key role in controlling a distinct virulome by an as yet unknown mechanism (Rampioni et al., 2016) and its expression is upregulated by AQ activation of the *pqsABCDE* operon, transcription of *pqsE* and virulence factors specifically controlled by PqsE was investigated during infection with PAO1 and Δ*pqsA* in the presence and absence of exogenous PQS. Samples were collected at 3 hpi and transcription of *pqsE* alongside that of *pqsA* and those of *mexG* and *lecA*, which are specifically controlled by PqsE (Rampioni et al., 2016), was examined by RT-PCR. The *mexG* gene codes for a multidrug efflux pump component and *lecA* for a cytotoxic galactophilic lectin known to inhibit growth, ciliary beating frequency, and the morphology of human respiratory cells (Bajolet-Laudinat et al., 1994; Aendekerk et al., 2005; Chemani et al., 2009). In agreement with the comparable growth of PAO1 and Δ*pqsA* in the presence and absence of exogenous PQS (Figure 5B), expression of the housekeeping gene *oprL* was consistent during infection with both strains in the presence and absence of PQS (Figure 6).

Transcription of *pqsA* was observed in the PAO1-infected samples while levels increased on provision of exogenous PQS (Figure 6). As expected, the *pqsA* transcript, because of the nature of the gene deletion, was not detected in Δ*pqsA-*infected cultures in the absence or presence of PQS (Figure 6). Interestingly, the *pqsE* and *mexG* transcripts were clearly expressed in the absence of PQS not only in the PAO1 but also in the Δ*pqsA-*infected cultures. In both instances, exogenous PQS increased expression of both target genes although levels were consistently lower in the case of Δ*pqsA* (Figure 6 and Figure S6B). Limited *lecA* expression could be detected in PAO1 and Δ*pqsA* infections. As above, *lecA* transcription was lower in the Δ*pqsA* infection and was upregulated by exogenous PQS in both WT and Δ*pqsA* infections (Figure 6). These results support AQ-independent transcription of *pqsE* and PqsE-controlled virulence factors during PAO1 infection of Calu-3-ALI cultures which can be upregulated by endogenous AQ production and exogenous PQS.

## Discussion

**Figure 7:**
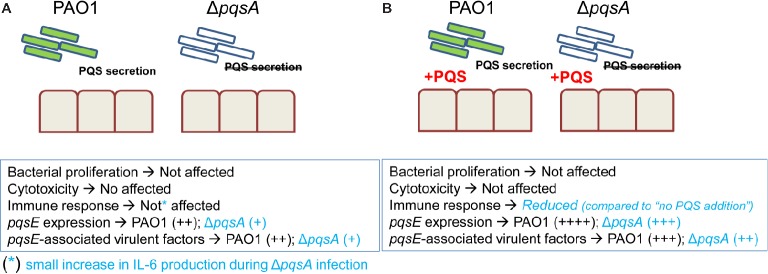
Overview of main findings of this study. In the absence and presence of exogenous PQS, growth of PAO1 and Δ*pqsA* is not affected and both strains caused similar cytotoxicity and cytokine production. Addition of exogenous PQS decreases cytokine production in response to infection with PAO1 and Δ*pqsA*. This observation indicates PQS could facilitate *P. aeruginosa* chronic infection by reducing inflammation. Basal expression of *pqsE* and *pqsE*-controlled virulence factors in the absence of PQS occurs during infection with levels being increased in the presence of endogenous AQs (PAO1 infection compared to Δ*pqsA* infection) and exogenous PQS (PAO1 and Δ*pqsA* infections performed in the presence and absence of PQS).

Previous work has uncovered a role for AQ-dependent QS in the control of *P. aeruginosa* virulence and the inhibition of immune responses by human and murine immune cells (Hooi et al., 2004; Skindersoe et al., 2009; Kim et al., 2010a,b). In the present study, we have demonstrated that: 1) AQs are produced during infection of differentiated bronchial epithelial cells with *P. aeruginosa* in a *pqsA*-dependent manner (Figure 1); but, surprisingly, the lack of AQ production does not influence bacterial growth (Figure 2A), cytotoxicity (Figures 2B,C), or immunostimulatory activity (Figure 3); 2) addition of exogenous PQS reduced the production of pro-inflammatory cytokines in response to *P. aeruginosa* infection (Figure 5A) without affecting bacterial growth (Figure 5B); and 3) expression of *pqsE* and PqsE-controlled virulence factors occurs in the absence of AQs during *P. aeruginosa* infection of HBECs (Figure 6). A graphical overview of these findings is shown in Figure 7.

The similar characteristics of PAO1 and Δ*pqsA* infections in the absence and presence of exogenous PQS suggest that virulence factors not regulated by AQs may be responsible for causing cellular damage during infection. These could include binding of *P. aeruginosa* type IV pili to N-glycans at the apical surface and the subversion of polarity facilitated by the binding of flagella to heparan sulfate proteoglycans that are enriched at the basolateral compartment of the epithelium (Bucior et al., 2010; Bucior et al., 2012). One study reported that PAO1 also induces airway epithelial apoptosis by promoting gap junctional communication through increased expression of the gap junction protein connexin 43 (Cx43) (Losa et al., 2014). Cx43 upregulation required flagellin expression (Losa et al., 2014). Our preliminary observations, in agreement with previous work (Losa et al., 2014), indicated that PAO1 and Δ*pqsA* both had a tendency to adhere to the junctions between adjacent cells. Both strains display a single polar flagellum (data not shown), which suggests that AQs do not interfere with bacterial swimming motility and bacterial attachment to the airway epithelium. In addition, PAO1 and Δ*pqsA* would similarly stimulate toll-like receptor 5, which is expressed by Calu-3 cells (Losa et al., 2014), and induce Cx43 upregulation. Hence, AQ-independent cytotoxic factors produced by *P. aeruginosa* may dominate the outcome of this acute infection model and mask the effects of AQ-controlled gene products on airway epithelium. Calu-3-ALI cultures may fail to provide selectivity for virulence traits mostly required for chronic infections such as adaptation to inflammatory conditions and biofilm development. Because of the direct effect of PQS on expression of HIF-1α (Legendre et al., 2012), it would be of interest to perform further studies under microaerobic conditions, such as those found in the CF lung (Schobert and Jahn, 2010), when PQS can still be synthesized and exert its influence on host adaptation to low oxygen through HIF-1α.

*P. aeruginosa* infection of Calu-3-ALI cultures promoted expression of pro-inflammatory cytokines, particularly TNF-α, IL-6, and IL-17C, at both mRNA and protein levels (Figures 3 and 5A). All three cytokines were downregulated by treatment with exogenous PQS (Figure 5A). These observations are in line with the effect of PQS on the NF-κB pathway (Kim et al., 2010a,b). Nevertheless, since PAO1 and Δ*pqsA* did not noticeably differ in their ability to induce cytokine production in this acute infection model in which AQ levels reached concentrations of 2.5 μM for HHQ and 2 μM for PQS, it is possible that PQS could only exert its immunomodulatory properties during PAO1 infection at higher concentrations (≥2 μM). A trend toward increased production of IL-6 and IL-17C was detected during infection with Δ*pqsA* compared to PAO1; this trend became significant in the case of IL-6 (Figure 5A). These results suggest that endogenous AQs might be approaching immunomodulatory concentrations during PAO1 infection of Calu-3-ALI cultures and that concentrations lower than 40 μM could be used to investigate the ability of PQS to regulate inflammation. This would be desirable as we do not know the physiological relevance of the 40 μM dose. PQS could act either by inhibiting NF-κB activation (Kim et al., 2010b) or by down modulating HIF-1α expression (Legendre et al., 2012). Future work involving infection models that incorporate macrophages and neutrophils will be used to further assess the immunomodulatory properties of AQs in the human system and their impact on bacteria survival. As part of the study, signaling pathways affected by the presence of AQs will be investigated.

Production of IL-17C by Calu-3-ALI cultures in response to *P. aeruginosa* is of particular interest. IL-17C is a member of the IL-17 family which is expressed by, and acts on, epithelial cells (Ramirez-Carrozzi et al., 2011). Thus, IL-17C would initiate a priming loop at the epithelial barrier that may have important consequences for establishment of infection and ensuing inflammatory responses, through the production, among others, of antimicrobial peptides (Ramirez-Carrozzi et al., 2011). The importance of IL-17C during *P. aeruginosa* infection has been recently highlighted using a mouse model (Wolf et al., 2016). Reduced neutrophil and neutrophil-recruiting cytokines as well as increased host survival was reported in IL-17C-deficient animals, which is in agreement with inflammation having a negative impact on susceptibility to *P. aeruginosa* infection (Wolf et al., 2016).

Expression of the AQ-regulated gene *pqsE* and in turn the PqsE-controlled genes *mexG* and *lecA* in the presence and absence of AQs was investigated in this infection model. Based on our findings, the in-frame mutation of *pqsA* allows transcription of the other members of the *pqsA-E* operon (*pqsB*, *pqs*C, and *pqs*D, data not shown) in addition to *pqsE* although, as expected, at lower levels than in the case of PAO1. These results demonstrate that during infection of HBECs, *pqsE* transcription and, in turn, transcription of PqsE-controlled virulence factors such as lectin A (Bajolet-Laudinat et al., 1994; Diggle et al., 2006) and the multidrug efflux pump *MexGHI-OpmD* (Aendekerk et al., 2005), could be driven in the absence of AQ. This is the first description of *pqsE*, *mexG,* and *lecA* expression in the absence of AQ during infection. These findings are consistent with previous studies using bacterial cultures (Dotsch et al., 2012; Knoten et al., 2014) which demonstrated the existence of several transcription start sites for the *pqsABCDE* operon (Dotsch et al., 2012) and *pqsE* transcription under nutrient-limiting conditions (Knoten et al., 2014). Nevertheless, AQs produced endogenously and/or provided exogenously promote expression of *pqsE* during infection and, in turn, that of virulence factors that contribute to *P. aeruginosa* pathogenesis. These results are in agreement with a role for AQs in promoting *P. aeruginosa* virulence (Deziel et al., 2005; Rampioni et al., 2010; Dubern et al., 2015). The therapeutic potential of *pqs* system inhibitors (Starkey et al., 2014) would arise, at least in part, from their ability to disturb the amplification of PQS/HHQ-controlled virulence factor expression. The lack of differences between PAO1 and Δ*pqsA* with respect to growth and cytotoxicity in the Calu-3-ALI cultures was surprising and may be due to AQ-independent virulence factors dominating bacterial fitness during infection of HBECs in this system (see above). Alternatively, since virulence factors that are PqsE-dependent can still be expressed in a *pqsA* mutant, our data also imply that PQS-dependent but PqsE-independent virulence factors are also among the factors that are not required for HBEC infection. These are likely to include the subset of genes regulated by PQS that are independent of PqsR (Rampioni et al., 2016). In addition, establishing the importance of PQS as immunomodulator during *P. aeruginosa* infection will require models that facilitate the establishment of chronic infection and incorporate immune cells such and macrophages and neutrophils.

In summary, this study characterized *P. aeruginosa* infection of differentiated HBECs in the presence and absence of endogenous and exogenous AQs. Parameters investigated include bacterial growth, cytotoxicity, cytokine production, and expression of the AQ-regulated genes *pqsE* and PqsE-controlled virulence factors. Our results show that although AQ-dependent QS enhances virulence gene expression in this model, upregulation did not lead to increased pathogenesis probably because the traits required for bacteria fitness during infection of HBECs are AQ-independent. These findings also support a role for PQS as immunomodulator in the lung; however, the doses of PQS required for this effect were probably not reached under these *in vitro* conditions. These findings stress the need for infection models that support immune cell involvement and chronicity to fully dissect which aspects of *P. aeruginosa* virulence should be targeted when interfering with the *pqs* QS system.

## Author Contributions

Y-CL generated and characterized Δ*pqsA*. Y-CL, FH and AP performed and analyzed all infections for CFU, cytotoxicity, and cytokine production. ON and PT contributed to the protein microarray analysis. NH performed the analysis of AQs during infection. J-FD performed RT-PCR analysis of bacterial gene expression. SS, SM, and CB contributed to optimization of Calu-3-ALI cultures. Y-CL, LW, and JL performed confocal analysis. Y-CL, PW, MC, and LM-P designed the study and wrote the manuscript.

### Conflict of Interest Statement

The authors declare that the research was conducted in the absence of any commercial or financial relationships that could be construed as a potential conflict of interest.
