# Corrigendum: Contribution of the Alkylquinolone Quorum-Sensing System to the Interaction of *Pseudomonas Aeruginosa* With Bronchial Epithelial Cells

**DOI:** 10.3389/fmicb.2019.00314

**Published:** 2019-02-21

**Authors:** Yi-Chia Liu, Farah Hussain, Ola Negm, Ana Carolina Paiva, Nigel Halliday, Jean-Frédéric Dubern, Sonali Singh, Sirina Muntaka, Lee Wheldon, Jeni Luckett, Paddy Tighe, Cynthia Bosquillon, Paul Williams, Miguel Cámara, Luisa Martínez-Pomares

**Affiliations:** ^1^School of Life Sciences, University of Nottingham, Nottingham, United Kingdom; ^2^Microbiology and Immunology Department, Faculty of Medicine, Mansoura University, Mansoura, Egypt; ^3^Centre for Biomolecular Sciences, University of Nottingham, Nottingham, United Kingdom; ^4^School of Medicine, University of Nottingham, Nottingham, United Kingdom; ^5^School of Pharmacy, University of Nottingham, Nottingham, United Kingdom

**Keywords:** bronchial epithelial cells, *Pseudomonas aeruginosa*, quorum sensing, inflammation, pseudomonas quinolone signal

An author name was incorrectly spelled as “Ana Pavia.” The correct spelling is “Ana Carolina Paiva.”

The authors apologize for this error and state that this does not change the scientific conclusions of the article in any way. The original article has been updated.

## Conflict of Interest Statement

The authors declare that the research was conducted in the absence of any commercial or financial relationships that could be construed as a potential conflict of interest.

