# Supplementary material for: Contribution of the Alkylquinolone Quorum-Sensing System to the Interaction of Pseudomonas aeruginosa With Bronchial Epithelial Cells
Source: Front Microbiol. 2018 Dec 18;9:3018. doi: 10.3389/fmicb.2018.03018 (PMC6305577; doi:10.3389/fmicb.2018.03018)
Supplement: Supplementary file 1 [file Data_Sheet_1_v1.PDF]

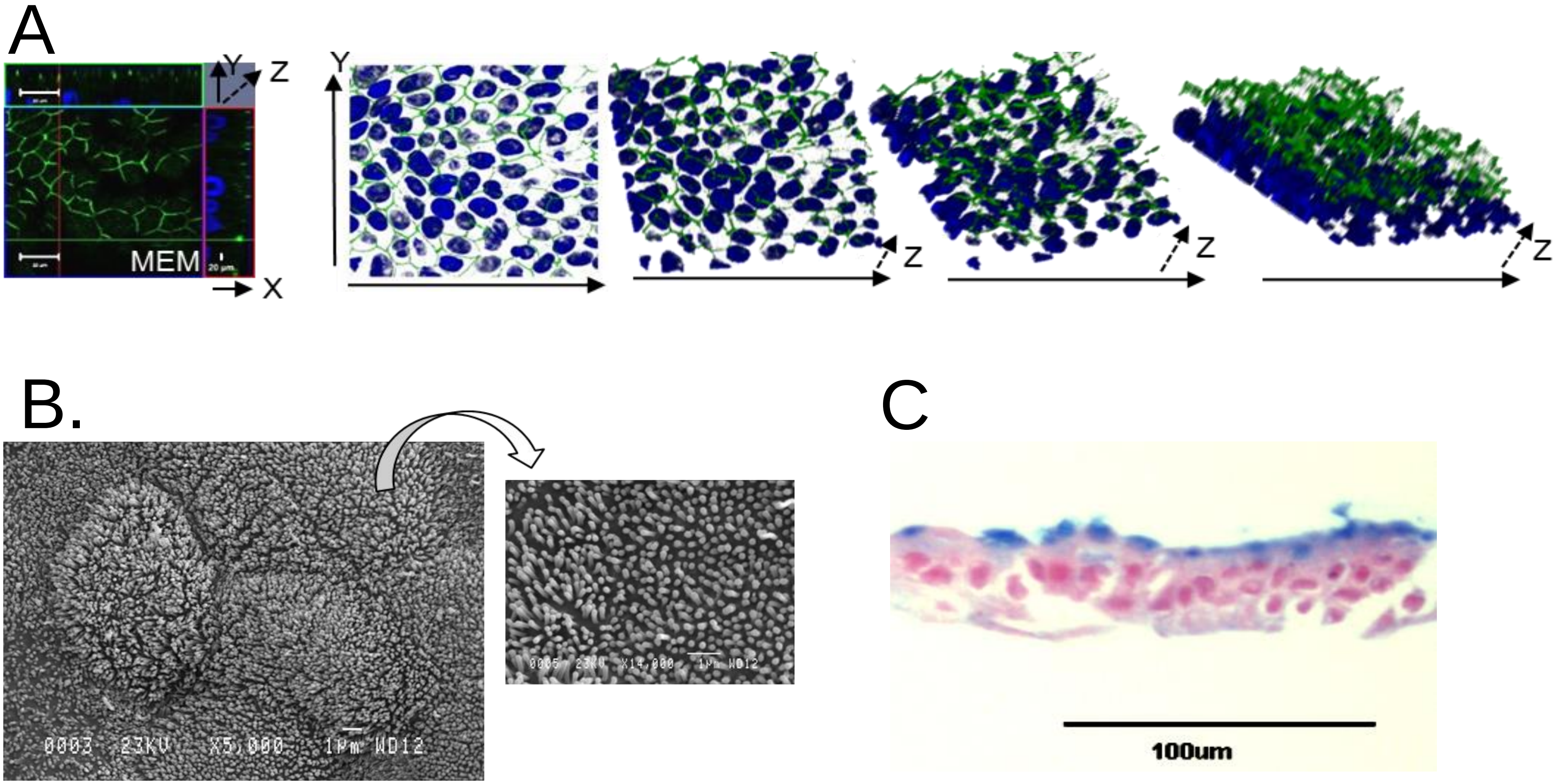

Fig. S1. *Liu et al*

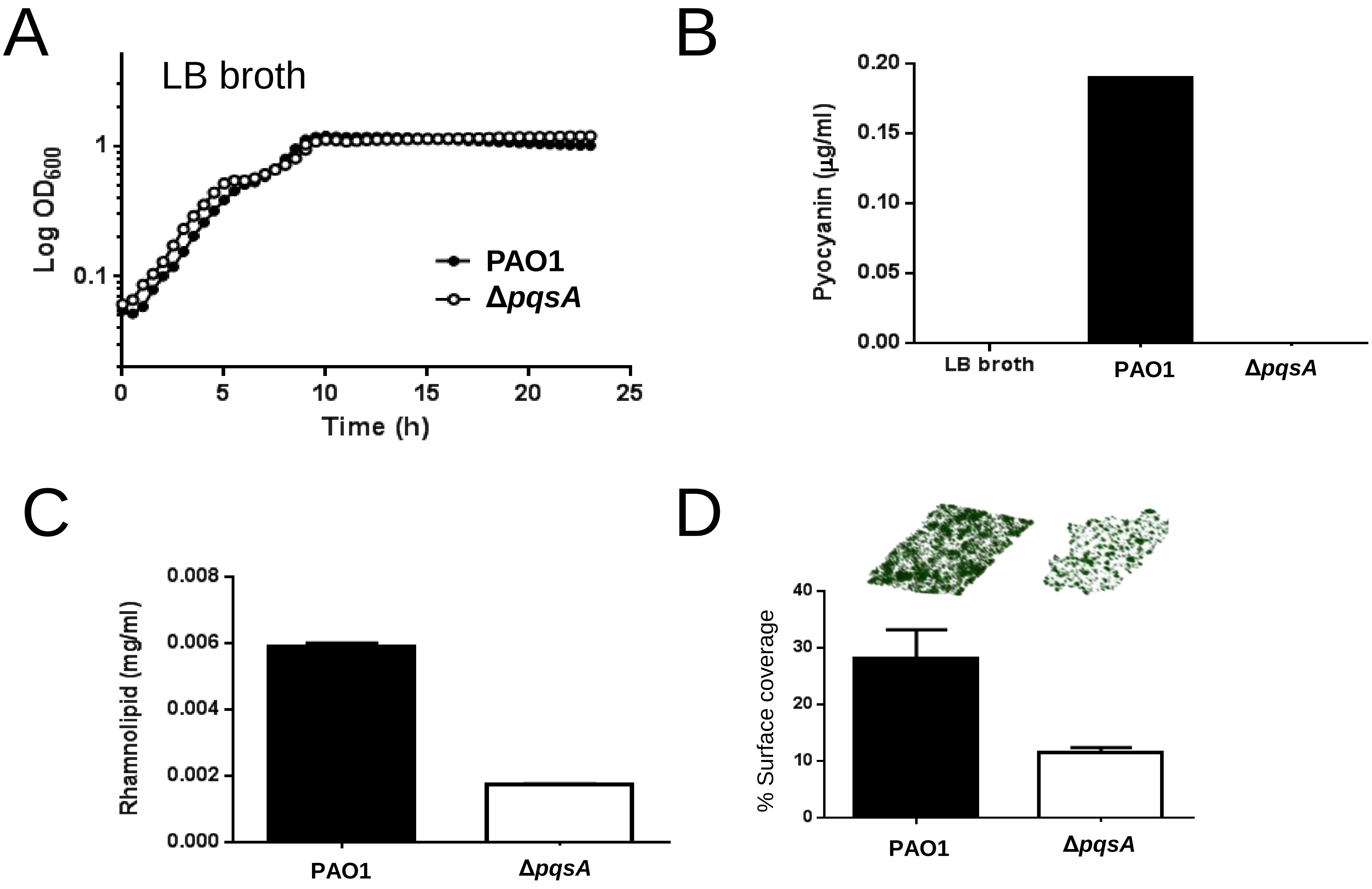

Fig. S2. *Liu et al.*

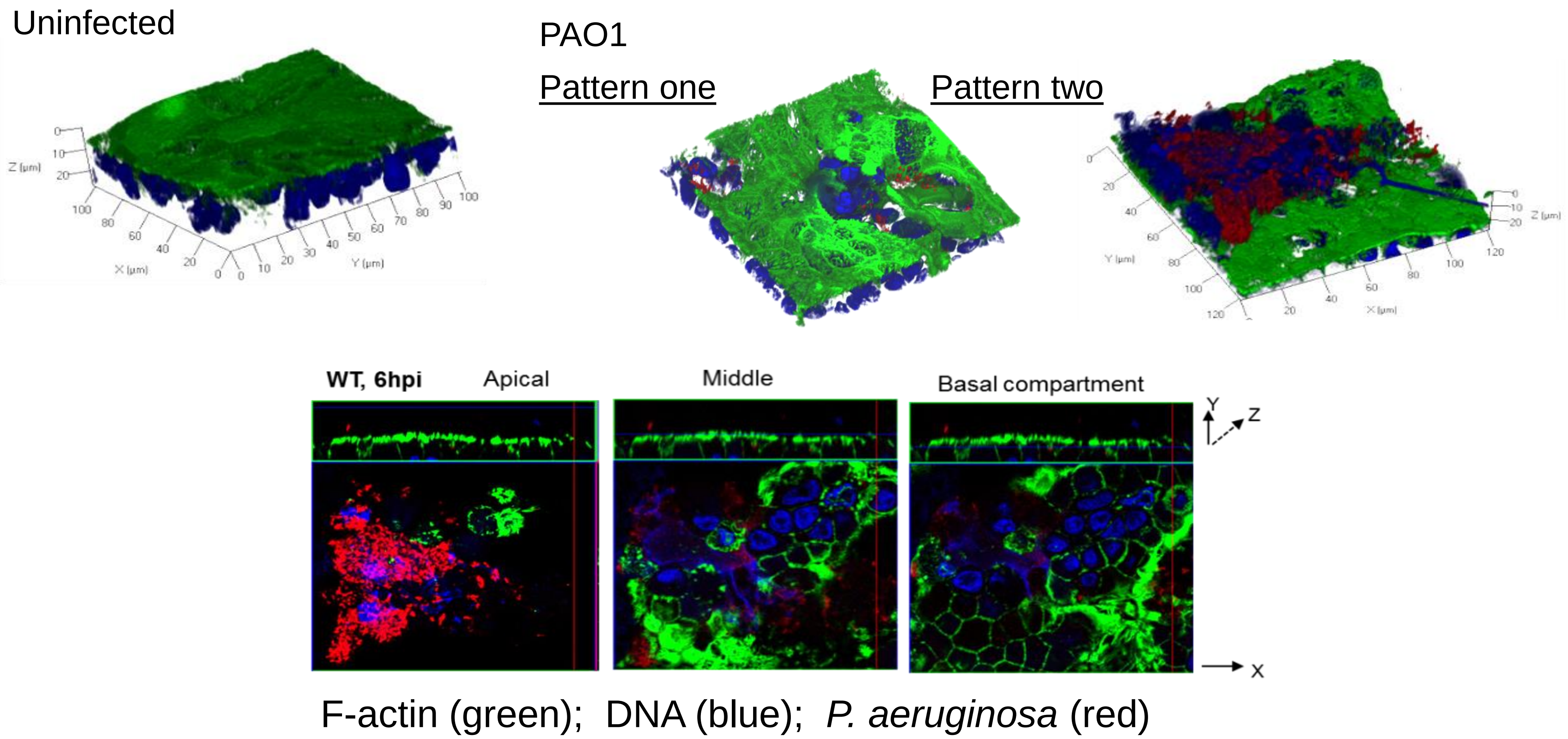

Fig. S3. *Liu et al.*

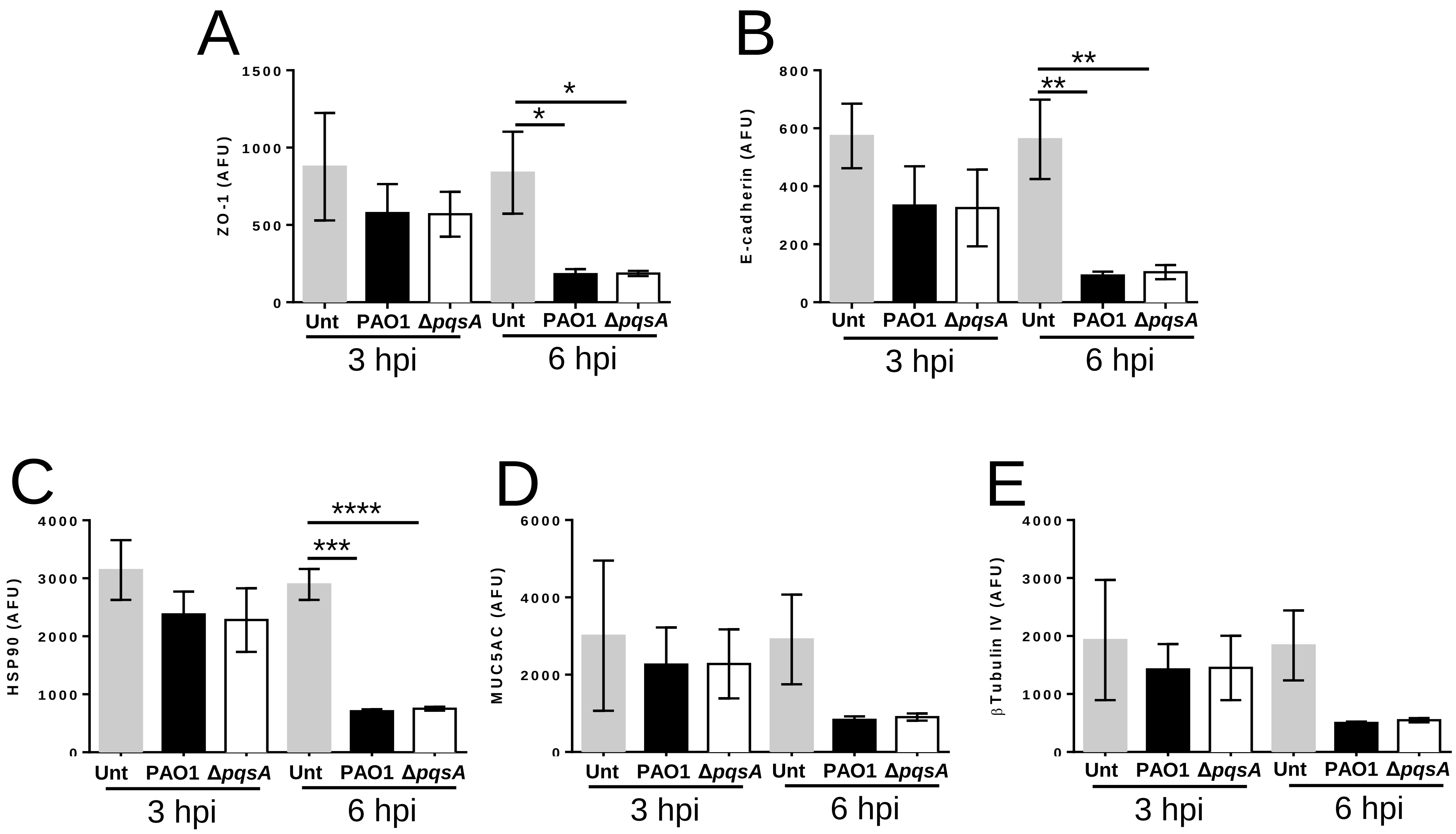

Fig. S4. *Liu et al.*

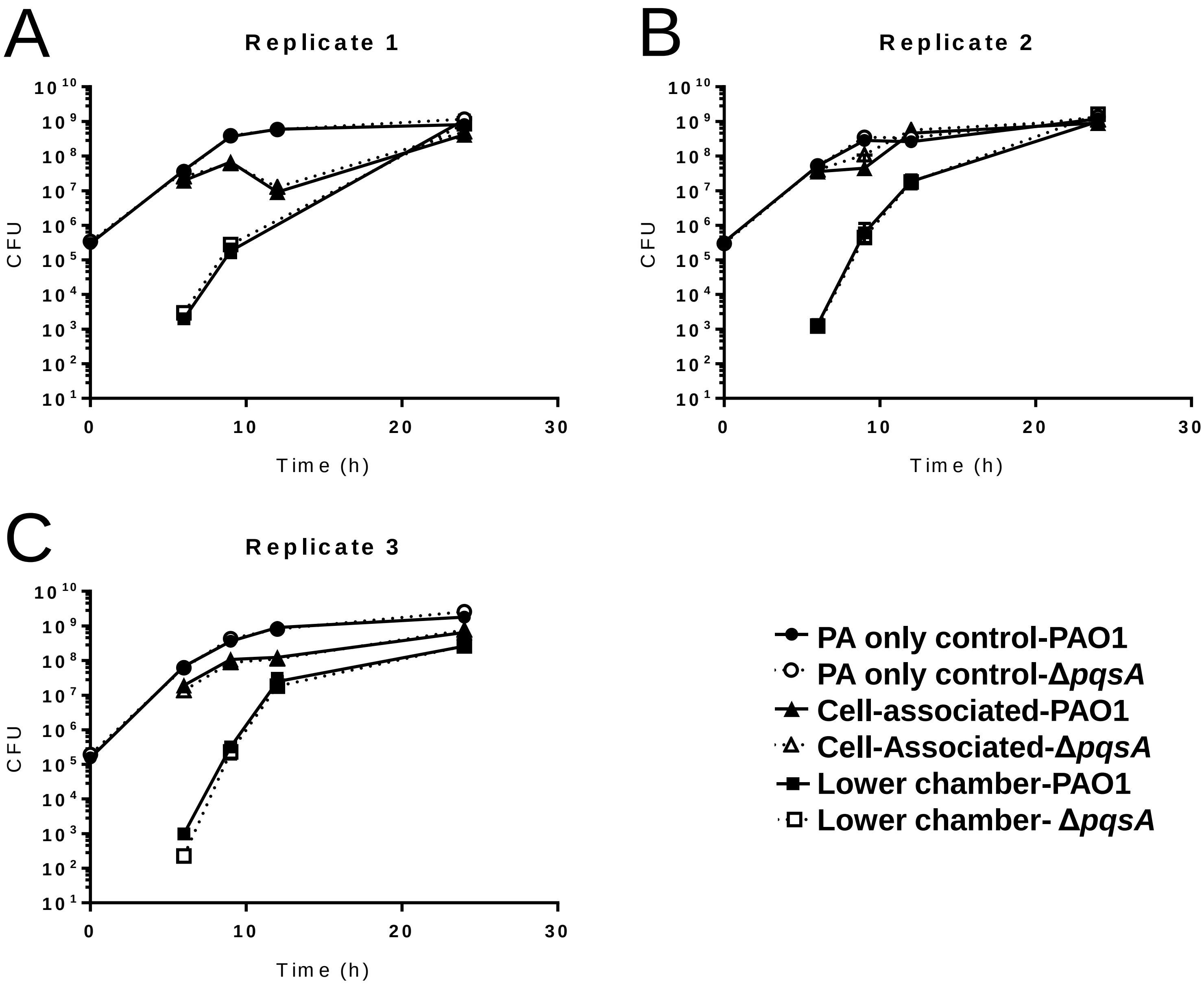

Fig. S5. *Liu et al.*

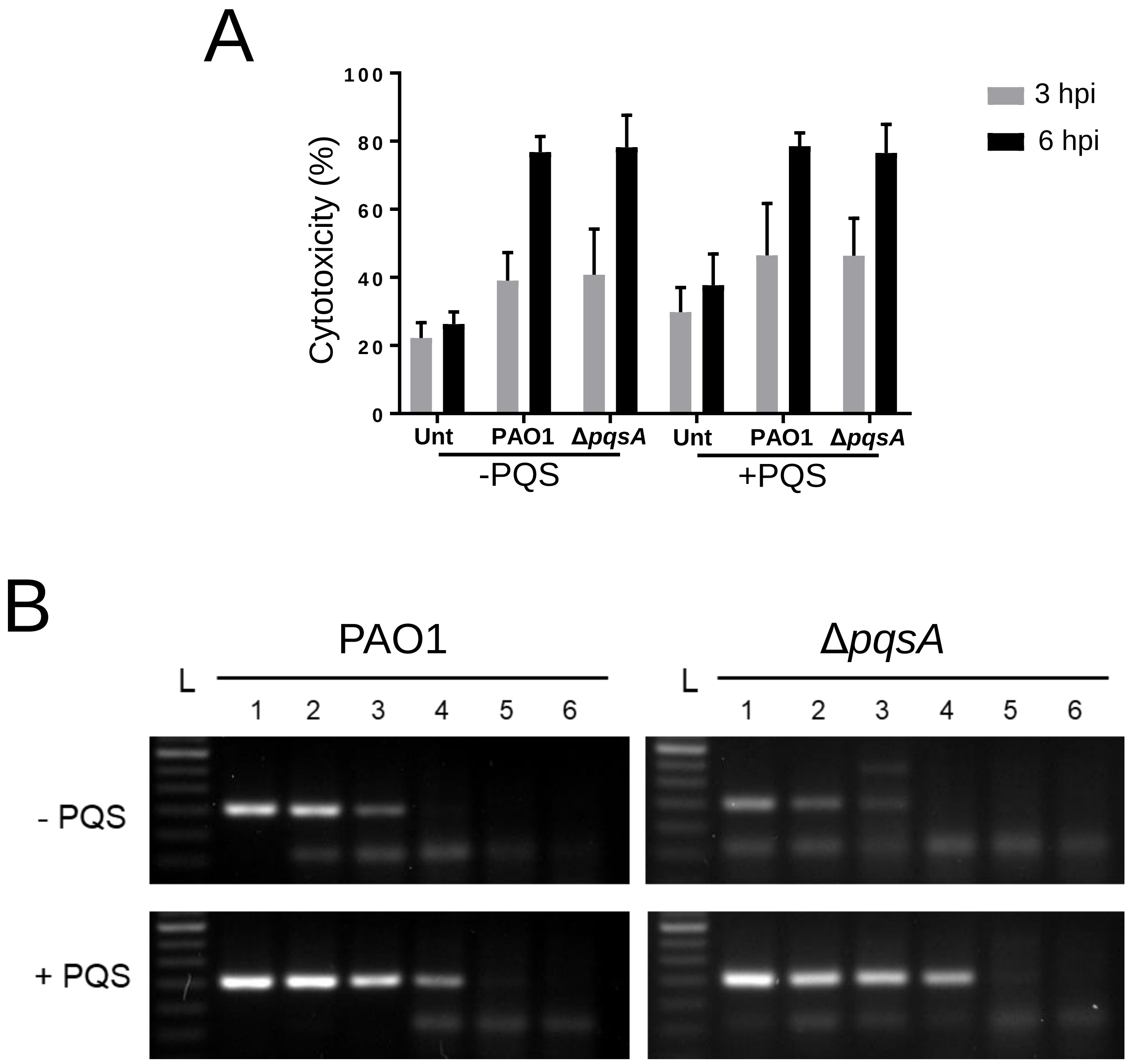

Fig. S6. *Liu et al.*
