# Supplementary material for: Contribution of the Alkylquinolone Quorum-Sensing System to the Interaction of Pseudomonas aeruginosa With Bronchial Epithelial Cells
Source: Front Microbiol. 2018 Dec 18;9:3018. doi: 10.3389/fmicb.2018.03018 (PMC6305577; doi:10.3389/fmicb.2018.03018)
Supplement: Supplementary file 2 [file Data_Sheet_2_v1.PDF]

Contribution of the alkylquinolone quorum sensing system to the interaction of *Pseudomonas aeruginosa* with bronchial epithelial cells.

Supplementary materials and methods

Calu-3 cell differentiation and physiological properties

1. Calu-3 cell culture and differentiation

Calu-3 cell culture handling and differentiation were described previously (Grainger et al., 2006). Calu-3 human bronchial epithelial cells (ATCC no. HTB-55) were purchased from the American Type Culture Collection (Rockville, USA) and used between passage 19 and 35. For differentiation, cells were seeded on 0.4 µm pore size, 12-well transwell polyester inserts (Corning Life Sciences, the Netherlands) at a density of 100,000 cells per well in 500 µl of complete culture medium with 1 ml of medium in the basal chamber. After overnight culture at 37°C, 5% CO_2, the medium in the insert was aspirated to enable the cells to differentiate at the air-liquid interface (ALI). The medium in the lower chamber was changed every other day up to 21 days.

2. Formation of tight barrier junctions (Fig. S1A)

Zonula occludin 1 (ZO-1) is prominently expressed in airway epithelial cells near the apical compartment as part of tight junction (TJ) proteins to form an effective barrier. Formation of tight junctions in the differentiated Calu-3-ALI was assessed by the expression of ZO-1. Three-week old Calu-3-ALI were fixed with 4% paraformaldehyde (16% paraformaldehyde, EM grade, Electron Microscopy Sciences, USA), washed with PBS, permeabilised with 0.15% Triton X-100 (Sigma-Aldrich, UK). Non-specific binding was blocked with the blocking solution (5% donkey serum, Sigma-Aldrich, in PBS), stained with anti-ZO-1 antibody (Life Technologies, USA) followed by secondary antibody donkey anti-mouse IgG conjugated to Alexa-Fluor 488 (Jackson ImmunoResearch, USA). Cells were counter stained with Hoechst 33342 (Life Technologies, USA). After staining, samples were mounted with Vectashield HardSet Mounting Medium (Vector Laboratories, UK). Images were acquired by confocal laser scanning microscopy (Zeiss LSM 700).

3. Visualisation of cilial protrusions (Fig. S1B) and mucus production (Fig. S1C)

Calu-3-ALI surface structures were visualised using scanning electron microscopy. Calu-3 cells cultured on transwell inserts at ALI for 21 days were fixed with 3% glutaraldehyde (Agar Scientific, UK) in 0.1 M sodium cacodylate buffer (Agar Scientific, UK) at 4°C. Samples were then post-fixed *in situ* with 1% osmium tetroxide (Agar Scientific, UK), dehydrated in serial concentrations of ethanol, and air-dried in a fume hood overnight. Once dry, inserts were cut out, mounted on inert supports and gold-sputter coated. Samples were visualised using a Jeol JSM840 scanning electron microscope with an ISS Iscan digital frame grabber. For histological staining, Calu-3-ALI transwell inserts were fixed with 4% paraformaldehyde, embedded in paraffin wax using a Leica TP1020 automated tissue processor and 5 µm thin cross-sectioned by a Leica RM2145 microtome. The air-dried cross-sections were rehydrated with decreasing concentrations of ethanol and stained with haematoxylin and eosin (Raymond A Lamb Laboratory Supplies, UK), Alcian blue/Mayer's acid (Sigma-Aldrich, UK), or Alcian blue/fast acid stain (Sigma-Aldrich, UK). Following staining, sections were dehydrated by passing through increasing concentrations of ethanol, dried, and mounted using DPX mounting medium (Raymond A Lamb Laboratory

Supplies, UK). Mounted sections were viewed using a Zeiss Axioplan light microscope and images were captured with a Q Imaging MicroPublisher 5.0 RTV camera along with Openlab software.

Phenotypic characterisation of PAO1-L* and *ΔpqsA

1. Bacterial growth in LB medium (Fig. S2A)

Bacterial growth was measured under static conditions. *P. aeruginosa* cultures were grown in a 96-well plate and OD_{600} was read every 30 min for 24 h using a microplate reader (Infinite® M1000 PRO, TECAN, UK). Samples were prepared in triplicate.

2. Pyocyanin production (Fig. S2B)

Pyocyanin was quantified using the colorimetric method described by Essar *et al*(Essar et al., 1990). Pyocyanin concentrations, presented as μg per ml, were determined by multiplying the absorbance value at A_{520} after adjusting for growth (OD_{600}) by 17.072 (Essar et al., 1990).

4. Rhamnolipids production (Fig. S2C)

Quantification of rhamnolipids was done using the orcinol method essentially as described by Christova et al. (Christova et al., 2004) Rhamnolipid concentrations were calculated as mg per ml. Each sample was prepared in triplicate.

5. Analysis of biofilm formation (Fig. S2D)

UV-sterilised glass slides were placed in petri-dishes inoculated *P. aeruginosa* cultures in RMPI-1640 (Sigma-Aldrich, UK) and started with an overnight culture in RPMI-1640 diluted to OD_{600} 0.05 in RPMI-1640. Cultures were incubated at 37°C with shaking at 60 rpm for 72 h. Glass slides were washed 3 times with PBS, rinsed with distilled water once and stained with SYTO9 and propidium iodine (FilmTracer Live/Dead Biofilm Viability Kit; Life Technologies, USA). Images were acquired by confocal laser scanning microscope (Zeiss LSM 700). Green-channel images were acquired using 488 nm excitation and 515 nm emission; red-channel images were collected using 594 nm excitation and emission at 625 nm. Biofilm surface coverage percentage was analysed using software ImageJ and the means of surface coverage were taken from 7 images taken from different fields on a glass slide.

***Analysis of* P. aeruginosa *infection of Calu-3-ALI cultures by confocal microscopy.* (Fig. S3)**

To visualise the distribution of *P. aeruginosa* PAO1 cells on Calu-3-ALI, immunofluorescence labelling was performed. Calu-3-ALI cultures infected with PAO1 were fixed, permeabilised, blocked, and bacteria detected with primary antibody rabbit polyclonal anti-Pseudomonas antibody (Abcam, USA) and secondary antibody donkey anti-rabbit IgG conjugated to Alexa-Fluor 674. F-actin was stained with phalloidin-Alexa 488 and nuclei counterstained with Hoechst 33342 (Life Technologies, USA). Images were acquired using a Zeiss LSM 700 laser scanning microscope and images analysed using software Zen 2009 (Zeiss. Germany).

Supplementary figure legends

Fig. S1

Calu-3-ALI cultures display the characteristics of differentiated bronchial epithelial cells. Calu-3-ALI cultures express tight junction protein ZO-1 (green) (A), presented cilia (B) and produced mucus (blue) (C) at the apical surface.

Fig. S2

Phenotypic characterisation of Δ*pqsA*. (A) Deletion of *pqsA* in PAO1 does not affect bacterial growth in LB broth. (B-C) Production of pyocyanin (B) and rahmnolipids (C) by Δ*pqsA* was substantially reduced in comparison with PAO1. Rhamnolipid production in Δ*pqsA* was 3 fold lower than WT. (D) Biofilm development by Δ*pqsA* is reduced in comparison with PAO1.

Fig. S3

Analysis of *P. aeruginosa* growth on Calu-3-ALI cultures using confocal microscopy. Representative images obtained by laser scanning confocal microscopy showed different patterns of PAO infection (pattern 1: low bacterial growth and low cytoxicity; and pattern 2: high bacterial growth and high cytotoxicity) in Calu-3-ALI cultures.

Fig. S4

PAO1 and Δ*pqsA* were similarly cytotoxic for Calu-3-ALI cultures. PAO1 and Δ*pqsA* caused significant loss of ZO-1 (*, p<0.05; *, p<0.05) (A), E-cadherin (**, p<0.005; **, p<0.005) (B) and HSP90 (***, p<0.0005; ****, p<0.0001) (C) in Calu-3-ALI cultures in comparison to untreated cultures. Similar loss of MUC5AC (D) and β-tubulin (E) in Calu-3-ALI cultures by WT and Δ*pqsA* infection at 6 hpi were also identified, although the differences with untreated cells were not statistically significant. *P*-value was determined by one-way ANOVA. *, p<0.05; **, p<0.005; ***, p<0.001.

Fig. S5.

Growth of PAO1 and Δ*pqsA* at low MOI infection of Calu-3-ALI cultures.

Calu-3-ALI cultures were infected with PAO1 or Δ*pqsA* PAO1 at MOI 0.5 and cell-associated bacteria, bacteria in the lower chamber and bacteria in the cell-free wells were quantified at 6, 9, 12 and 24 hpi. No significant growth differences between PAO1 and Δ*pqsA* were observed in any of the assays. This assay was repeated three times (Replicates 1, 2 and 3).

Fig S6.

Effect of exogenous PQS on cytotoxicity and *pqsE* transcription.

(A). Exogenous PQS does not influence cellular cytotoxicity in response to PAO1 and Δ*pqsA* infection. Calu-3 cells exposed to PQS (40 µM) or DMSO were infected with PAO1 or Δ*pqsA* at MOI 50 for 3 and 6 hpi. Supernatants were collected and tested for LDH levels. (B). Analysis of *pqsE* transcription with and without addition of PQS in Calu-3-ALI cultures infected with PAO1 and Δ*pqsA* using RT-PCR. cDNA was synthesised from 300 ng (lane 1), 120 (lane 2), 30 (lane 3), 12 (lane 4), 6 (lane 5) and 0.6 (lane 6) of total RNA extracted from infected cells.

Supplementary references

CHRISTOVA, N., TULEVA, B., LALCHEV, Z., JORDANOVA, A. & JORDANOV, B. 2004. Rhamnolipid biosurfactants produced by Renibacterium salmoninarum 27BN during growth on n-hexadecane. *Z Naturforsch C,* 59**,** 70-4.

ESSAR, D. W., EBERLY, L., HADERO, A. & CRAWFORD, I. P. 1990. Identification and characterization of genes for a second anthranilate synthase in Pseudomonas aeruginosa: interchangeability of the two anthranilate synthases and evolutionary implications. *J Bacteriol,* 172**,** 884-900.

FLETCHER, M. P., DIGGLE, S. P., CAMARA, M. & WILLIAMS, P. 2007. Biosensor-based assays for PQS, HHQ and related 2-alkyl-4-quinolone quorum sensing signal molecules. *Nat Protoc,* 2**,** 1254-62.

GRAINGER, C. I., GREENWELL, L. L., LOCKLEY, D. J., MARTIN, G. P. & FORBES, B. 2006. Culture of Calu-3 cells at the air interface provides a representative model of the airway epithelial barrier. *Pharm Res,* 23**,** 1482-90.

GRANT, S. G., JESSEE, J., BLOOM, F. R. & HANAHAN, D. 1990. Differential plasmid rescue from transgenic mouse DNAs into Escherichia coli methylation-restriction mutants. *Proc Natl Acad Sci U S A,* 87**,** 4645-9.

SIMON, R., PRIEFFER, U. & PUHLER, A. 1983. A Broad Host Range Mobilization System for In Vivo Genetic Engineering: Transposon Mutagenesis in Gram Negative Bacteria. *Nature Biotechnology,* 1**,** 784-791.

VOISAD, C., BULL, C. T., KEEL, D., LAVILLE, J., MAURHOFER, M., SCHNIDER, U., DFEAGO, G. & HAAS, D. 2007. Biocontrol of root diseases by Pseudomonas fluorescens CHA0: current concepts and experimental approaches. *Molecular Ecology of Rhizosphere Microorganisms.* Wiley-VCH Verlag GmbH.

Supplementary Table 1

Table S1. Bacterial strains and plasmids used in this study

Strain/plasmid	Relevant characteristics	Reference or origin
**Strain**		
E. coli		
DH5α	Cloning strain; F^- *endA1 hsdR17 supE44 thi-1 recA1 gurA96 relA1* Δ(*lacZYA-argF*)U169 *deoR* λ(φ80*lac*ZΔM15)	(Grant et al., 1990)
S17-1λ*pir*	Conjugative strain for suicide plasmids	(Simon et al., 1983)
P. aeruginosa		
PAO1	Wild-type *P. aeruginosa* from Université de Lausanne, Switzerland. Also known as PAO1-L	Dieter Haas collection
Δ*pqsA*	*pqsA* mutant in PAO1-L background	This study
PAO1 Δ*pqsA CTX-lux::pqsA*	Chromosomal deletion of the *pqsA* gene in PAO1 containing CTX::P_{pqsA}-*lux*	(Fletcher et al., 2007)
**Plasmids**		
pBluescript-II KS+	Cloning vector; ColE1 replicon; Amp^R	Agilent Technologies, US
pME3087	Suicide vector, polylinker of pMMB67, ColE1-replicon; Tc^R	(Voisad et al., 2007)
pYCL1	pME3087 derivative for *pqsA* in-frame deletion; Tc^R	This study

Amp^R, ampicillin resistant; Tc^R, tetracyclin resistant

Supplementary Table 2. Primers used for Q-PCR.

Primer (Accession number)	Primer Sequences	Amplicon length (nucleotides)
**NM_000194.2**		
HPRT_f	5’-GTAATGATCAGTCAACGGGGGAC-3’	177
HPRT_r	5’-CCAGCAAGCTTGCAACCTTAACCA-3’	
**NM_000758.3**		
GM-CSF_f	5’-TCTVAGAAATGTTTGACCTCC-3’	98
GM-CSF_r	5’-GCCCTTGAGCTTGGTGAG-3’	
**NM_000600.3**		
IL-6_f	5’-GATGAGTACAAAAGTCCTGATCCA-3’	130
IL-6_r	5’-CTGCAGCCACTGGTTCTGT-3’	
**NM_000594.3**		
TNFaH_f	5’-GACAAGCCTGTAGCCCATGT-3’	105
TNFaH_r	5’-TCTCAGCTCCACGCCATT-3’	
**NM_000584.3**		
IL-8_h_f	5’-AGACAGCAGAGCACACAAGC-3’	62
IL-8_h_r	5’-ATGGTTCCTTCCGGTGGT-3’	
**NM_013278.3**		
IL-17c_f	5’-CCCTCAGCTACGACCCAGT-3’	126
IL-17c_r	5’-CTTCTGTGGATAGCGGTCCT-3’	

Supplementary Table 3. Primers used in Reverse Transcription-PCR.

Primer name	Sequence
*pqsE*RT For	5’-TGTTGAGGCTTTCGGCT-3’
*pqsE*RT Rev	5’-TGATCAGCCAGTAGTGCA-3’
*pqsA*RT For	5’-TCGACGATTTCTCGCTGG-3’
*pqsA*RT Rev	5’-CTGTTGCCCATGCCATAGC-3’
*oprL*RT For	5’-GAAATGCTGAAATTCGGCAAAT-3’
*oprL*RT Rev	5’-TGTCGTACTCGAAGTAG-3’
*mexG*RT For	5’-ATCTGCCTGGCCCTGATG-3’
*mexG*RT Rev	5’-AGAAGGTGTGGACGATGAGG-3’
*lecA*RT For	5’-ATCTACAATCCGGGCGATGT-3’
*lecA*RT Rev	5’-TGCACCCTGGACATTATTGG-3’
